# O04 Differences in public knowledge and attitudes towards antimicrobial resistance and antibiotic use across the four UK nations

**DOI:** 10.1093/jacamr/dlaf118.004

**Published:** 2025-07-14

**Authors:** Ellie Gilham, Kate Duxbury, Donna M Lecky

**Affiliations:** Antimicrobial Resistant and Healthcare-Associated Infection Division, United Kingdom Health Security Agency, London, UK; Ipsos (Market Research) Ltd, London, UK; Antimicrobial Resistant and Healthcare-Associated Infection Division, United Kingdom Health Security Agency, London, UK; Health Protection Research Unit in Healthcare Associated infections and Antimicrobial Resistance, Department of Medicine, Imperial College London, London, UK

## Abstract

**Background:**

Most antibiotics are prescribed in primary care where demand for consultations for common self-limiting infections is greatest and public actions may influence general practitioners prescribing decisions.

**Objectives:**

To highlight any differences in health-seeking behaviours, knowledge and attitudes towards AMR and antibiotic use between the four UK nations in order to identify how public messaging may need to be adapted for each nation.

**Methods:**

Ipsos MORI conducted a national UK wide survey in 2024. Quota sampling were used, with questionnaire responses weighted to ensure a representative sample. Pearson’s Chi-squared test was used to assess differences in proportions between the four UK nations.

**Results:**

Responses were obtained from 5914 participants (England: 51%, 3024/5914; Wales: 17%, 1027/5914; Northern Ireland (NI): 10%, 590/5914; Scotland: 22%, 1273/5914). The proportion of those who reported not having had an infection, and those who reported not having taken antibiotics in the previous 12-months was higher in England (18% and 67% respectively) compared to the other three nations (Wales: 14% and 62%; Scotland: 15% and 61%; Northern Ireland: 11% and 58% respectively; *P<*0.05). Knowledge of antibiotic use to treat specific diseases and attitudes towards AMR varied between nations. Those from NI were more likely to have seen, heard or read information on antibiotics in the previous 12-months compared to those from Scotland, Wales and England (55% versus 53%, 52% and 53% respectively; *P<*0.05). Reported behaviours also differed with respondents from Scotland, Wales, and NI more likely to have sought advice for their most recent illness from a pharmacy compared to respondents from England (14%, 15% and 20% versus 10%, *P<*0.05). Those from NI were more likely to have received a prescription *via* a telephone consultation with their GP (38%) compared to other nations (Scotland: 28%; Wales: 13%; England: 22%)). Finally, respondents from Scotland were least likely to request antibiotics from their GP (Scotland: 20% versus Wales, NI and England: 25%).

**Conclusions:**

Our findings highlight some significant differences in knowledge, attitudes and reported behaviours between UK nations. These differences highlight the need for tailored approaches to public engagement. They also outline potential areas of focus for public messaging and education strategies.

